# Mutations of the functional *ARH1* allele in tumors from *ARH1* heterozygous mice and cells affect ARH1 catalytic activity, cell proliferation and tumorigenesis

**DOI:** 10.1038/oncsis.2015.5

**Published:** 2015-06-01

**Authors:** J Kato, D Vekhter, J Heath, J Zhu, J T Barbieri, J Moss

**Affiliations:** 1 Cardiovascular and Pulmonary Branch, National Heart, Lung, and Blood Institute, National Institutes of Health, Bethesda, MD, USA; 2 Medical College of Wisconsin, Microbiology and Molecular Genetics, Milwaukee, WI, USA

## Abstract

ADP-ribosylation results from transfer of the ADP-ribose moiety of nicotinamide adenine dinucleotide (NAD) to an acceptor with ADP-ribose-acceptor content determined by the activities of ADP-ribosyltransferases, which modify the acceptor, and ADP-ribose-acceptor hydrolase (ARH), which cleave the ADP-ribose-acceptor bond. ARH1 was discovered as an ADP-ribose(arginine)protein hydrolase. Previously, we showed that *ARH1*-knockout and *ARH1* heterozygous mice spontaneously developed tumors. Further, *ARH1*-knockout and *ARH1* heterozygous mouse embryonic fibroblasts (MEFs) produced tumors when injected into nude mice. In tumors arising in *ARH1* heterozygous mice and MEFs, we found both loss of heterozygosity (LOH) of the *ARH1* gene and *ARH1* gene mutations. In the present report, we found that these mutant *ARH1* genes encode proteins with reduced ARH1 enzymatic activity. Moreover, MEFs transformed with *ARH1* mutant genes exhibiting different levels of ARH1 activity showed altered rates of proliferation, anchorage-independent colony growth in soft agar, and tumorigenesis in nude mice. MEFs transformed with the wild-type (WT) gene, but expressing low levels of hydrolase activity were also tumorigenic. However, transformation with the WT gene was less likely to yield tumors than transformation with a mutant gene exhibiting similar hydrolase activity. Thus, control of protein-ADP-ribosylation by ARH1 is critical for tumorigenesis. In the human cancer database, LOH and mutations of the *ARH1* gene were observed. Further, *ARH1* gene mutations were located in exons 3 and 4, comparable to exons 2 and 3 of the murine *ARH1* gene, which comprise the catalytic site. Thus, human *ARH1* gene mutations similar to their murine counterparts may be involved in human cancers.

## Introduction

Lung cancer is the most common cause of global cancer-related mortality.^1^ Adenocarcinoma, and to a lesser extent, squamous cell carcinomas, major subtypes of non-small-cell lung carcinoma have been shown to have mutations in critical regulatory genes.^1^ Gene mutations may change protein structure and enzymatic activity and may be associated with novel activities, for example, interactions with other cellular proteins, resulting in aberrant regulation of transcriptional programs.^2, 3^ Cellular kinases, for example, epidermal growth factor receptor and phosphatidylinositol 3 kinase, are frequently mutated and dysregulated in non-small-cell lung carcinoma.^3, 4^ High rates of somatic mutations in human lung adenocarcinoma have been found in regulatory genes such as Ras and p53.^1, 4, 5^

Post-translational modifications (for example, phosphorylation, acetylation and methylation) of proteins regulate cell proliferation and tumor development in mice.^6, 7, 8^ Histone methylation has been shown to affect non-small-cell lung carcinoma tumorigenesis.^3^ Phosphorylation, acetylation and ubiquitination of p53 influence protein stability and transcriptional activity with affects on cell proliferation and tumorigenesis.^6^ Protein poly-ADP-ribosylation by a family of poly (ADP-ribose) polymerases (PARPs) contributes to a variety of biological functions including carcinogenesis, chromosomal stability, regulation of chromatin structure, transcription, DNA repair and telomere homeostasis^9, 10, 11^ and was induced by genotoxic stress.^12^

Mono-ADP-ribosylation is a post-translational modification of proteins, in which the ADP-ribose moiety of β-nicotinamide adenine dinucleotide (NAD) is transferred to specific amino-acid residues in target proteins.^13^ Mono-ADP-ribosylation is used by some bacterial toxins to alter the activity of critical proteins in mammalian cells, thereby disrupting regulatory, biosynthetic or metabolic pathways.^14^ Cholera toxin, for example, ADP-ribosylated arginine in the α-subunit of the stimulatory guanine nucleotide-binding (G) protein of the adenylyl cyclase system, resulting in its activation and an increase in intracellular cAMP.^15^ ADP-ribosylation of G-actin at Arg177 by Salmonella enterica toxin (SpVB) inhibits ATPase activity and thereby prevents actin polymerization.^16, 17^ Other toxin ADP-ribosylatransferases (for example, *Clostridium limosum* exoenzyme C3, *Pertussis toxin, diphtheria toxin*) use different proteins, and in some instances, different amino acids (for example, asparagine, cysteine and modified histidine) as substrates.^13, 14, 18, 19^

In mammalian and avian systems, ADP-ribosylation of arginine appears to be a reversible modification of proteins.^20^ ADP-ribosyltransferases (ARTs) use β-NAD as the ADP-ribose donor and synthesize the α-anomeric ADP-ribosyl-arginine acceptor.^13, 19, 20^ Stereospecific ADP-ribosylarginine hydrolases (ARH) cleave α-ADP-ribose-arginine(protein), produced in the transferase-catalyzed reaction, to regenerate the unmodified arginine, which is then available for ADP-ribosylation on its guanidino moiety.^13, 16, 20, 21, 22^ Thus, ARHs complete an ADP-ribosylation cycle that could reversibly regulate the function of substrate proteins.^13, 20, 23^

Human neutrophil peptide 1 (HNP-1), a defensin found in human lung is believed to modulate the innate immune response.^24, 25^ ADP-ribosylated HNP-1 was isolated from the airways of patients with pulmonary disease (for example, asthma, pulmonary fibrosis).^24, 25^ ADP-ribosyl(arginine)-HNP-1 exhibited reduced antimicrobial and cytotoxic activities compared with native HNP-1, but with less of an effect on its function as a T-cell chemoattactant.^24, 25^ Other arginine ADP-ribosylations have been described. ADP-ribosylation of (arginine) P2X7 by ART2.2 leads to rapid apoptotic death of native murine T lymphocytes.^26^ Exposure of T cells to a low concentration of extracellular NAD lead to ADP-ribosylation of P2X7 by ART2.2, and resulted in cell shrinkage, fragmentation of DNA and exposure of phosphatidylserine.^26, 27^

ARH1 removes the regulatory ADP-ribose moiety from (arginine)proteins, regenerating unmodified acceptor proteins or molecules.^23, 28, 29^ ARH1 was identified as a 39-kDa protein in mammalian and avian tissues and cells, with human and mouse *ARH1* sharing structural similarities.^23, 28^ *In vitro*, human and avian ARH1 enzymatic activities were stimulated by dithiothreitol and Mg^2+^.^20, 30^ Mg^2+^ is believed to be necessary for the correct orientation of the substrates at the catalytic site.^30^

As noted, cholera toxin exerts its effects through the ADP-ribosylation of Gαs. The effects of cholera toxin, as evidenced by both ADP-ribose-arginine content and Gαs modification, were greater in *ARH1*-deficient mouse embryonic fibroblasts (MEFs) than in wild-type (WT) MEFs and were significantly reduced by overexpression of WT *ARH1* in *ARH1*-deficient MEFs.^31^ Similarly, fluid accumulation induced by cholera toxin in intestinal loops was greater in *ARH1*-deficient (*ARH1*KO) mice than in their WT counterparts, as was ADP-ribosylation of Gαs and ADP-ribosylarginine content of intestinal epithelial cells.^31^ These data support a role for ARH1 in the intoxication process.^31^

Recently, we reported increased tumorigenesis associated with *ARH1* deficiency.^32^ *ARH1*KO MEFs proliferated faster than their WT counterparts,^32^ and *ARH1*KO MEFs, but not their WT counterparts, formed colonies in soft agar and produced subcutaneous tumors in nude mice.^32^ Transfection of the WT *ARH1* gene into *ARH1*KO MEFs (*ARH1*KO+wt) prevented colony formation in soft agar and tumors following their subcutaneous injection in nude mice, whereas transformation of the KO MEFs with an inactive double-mutant (D60, 61A) *ARH1* gene had no effect.^32^ Consistent with these observations, *ARH1*KO mice spontaneously developed tumors more frequently than did their WT littermates. *ARH1* heterozygous MEFs and mice shared tumorigenic properties similar to their *ARH1*-deficient counterparts, which appeared to result from mutation or loss of the functioning *ARH1* allele.^32^

In some tumors from *ARH1* heterozygous mice or from nude mice after subcutaneous injection of *ARH1* heterozygous MEFs, an ARH1 protein band was observed by immunoblotting. In all instances, mutations in the *ARH1* gene were found in the tumor, but not in adjacent non-tumor tissue. Notably, no mutation was detected in complementary DNA (cDNA) from the *ARH1* heterozygous MEFs that had been injected. In all likelihood, an *ARH1* mutation in a small population of the heterozygous MEFs enabled them to proliferate more rapidly than did *ARH1* heterozygous MEFs containing a normal allele, thus giving rise to colonies in soft agar and tumors in nude mice.^32^ Mechanisms observed for inactivation of the active *ARH1* gene included loss of heterozygosity (LOH) and *ARH1* gene mutations.^32^

In this report, we characterize the functional effects of *ARH1* mutations identified in lung adenocarcinomas in *ARH1* heterozygous mice and in tumors in nude mice injected with *ARH1* heterozygous MEFs. These mutations were observed in exons 2 and 3 of the *ARH1* gene, which encode the region of the protein that forms the catalytic site.^23, 33^ The mutations did not always inactivate the protein. Using *ARH1*-deficient MEFs transfected with the mutated *ARH1* genes to measure of ARH1 function, different effects were seen with the different tumor-inducing gene mutations on proliferation, growth in soft agar and tumorigenesis in nude mice, suggesting that mutations in *ARH1*, in addition to effects on catalytic activity, could lead to conformational changes at the ARH1 active site, which alter other functions (for example, interaction with proteins). Given a possible role for the *ARH1* gene in cancer, we next used the Catalogue of Somatic Mutations in Cancer (COSMIC) database from Trust Sanger Institute (England)^34, 35^ to look for *ARH1* gene mutations and LOH in human cancer. Mutations were found in exons similar in location to those seen in our murine model. These data are consistent with a potential role for *ARH1* mutations in human, as well as murine cancer.

## Results

### Mutations of the *ARH1* gene in tumors

We reported that mutations in exons 2 and 3 of the *ARH1* gene were detected by in lung adenocarcinoma isolated from *ARH1* heterozygous mice and tumors in nude mice injected with *ARH1* heterozygous MEFs.^32^ Six mutations were identified in lung adenocarcinoma of *ARH1* heterozygous mice, and another eight mutations were found in tumors from nude mice injected with *ARH1* heterozygous MEFs (Tables 1 and 2). Mutation types included missense mutations resulting from single-base substitutions (12 of 14, 85.7%), and deletion mutations with frame shifts (2 of 14, 14.3%) (Tables 1 and 2, Supplementary Table 1). The most frequent mutations of the coding strand were A>G (5 of 14, 35.7%) and T>C (4 of 14, 28.6%) (Tables 1 and 2, Supplementary Table 1).

### Effects of mutations on ARH1 activity

**Figure 1:**
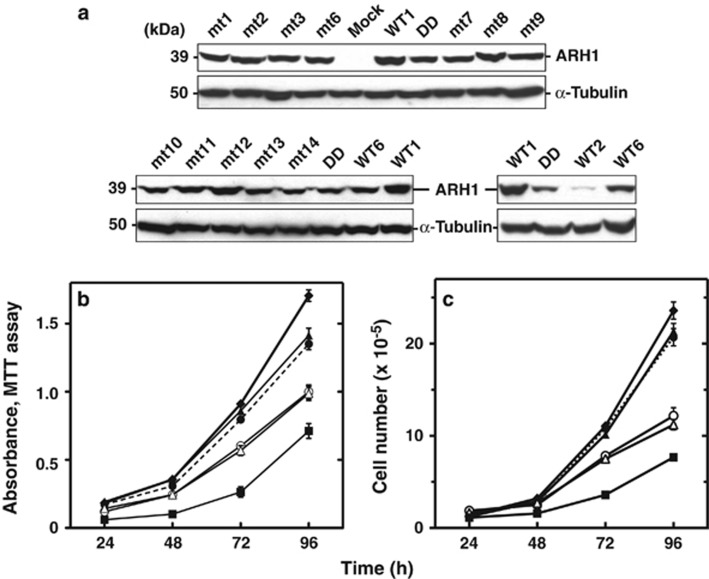
Proliferation of *ARH1*KO cells expressing a WT gene and mutant *ARH1* genes from *ARH1*^*+/−*^ mouse lung adenocarcinoma. (**a**) Immunoblotting with anti-ARH1 and anti-α-tubulin antibodies of proteins in cell lysates (50 μg) of *ARH1*KO MEFs transformed with *ARH1* WT genes and mutant genes. *ARH1*KO MEFs that expressed *ARH1* WT and mutant genes were used in the experiments (that is, ARH1 activity experiments, cell proliferation assays, soft agar colony forming assay, tumorigenesis studies in nude mice). (**b**) *ARH1*KO+WT (▪), *ARH1*KO Mock (♦), *ARH1*KO*+*mt1 (○), *ARH1*KO*+*mt2 (▴), *ARH1*KO+mt3 (●) and *ARH1*KO+mt6 (Δ) MEFs (5 × 10^3^) were seeded in 96-well plates and MTT assays were performed after growth for the indicated time. (**c**) *ARH1*KO+WT (▪), *ARH1*KO mock (♦), *ARH1*KO*+*mt1 (○), *ARH1*KO*+*mt2 (▴), *ARH1*KO+mt3 (●) and *ARH1*KO+mt6 (Δ) MEFs (1 × 10^5^) were seeded in 100 mm dishes and cell counting was performed after growth for the indicated time. Data are means±s.e.m. of values from six assays performed in three experiments. Pairwise comparison showed that *ARH1*KO Mock, +mt2, +mt3 and +WT were significantly different from *ARH1*KO+mt1, and +mt6 (all at *P*<0.001), and also all *ARH1*KO+mutations and *ARH1*KO mock were significantly different from *ARH1*KO+WT (all at *P*<0.001).

To determine the effects of mutations on ARH1 enzymatic activity, the *ARH1* mutant genes were placed in a mammalian expression vector and expressed in *ARH1*KO MEFs. We generated stably transformed *ARH1*KO MEFs with *ARH1* WT and mutant genes including mock (empty vector). Similar expression levels of protein were detected by western blots (Figure 1a). Surprisingly, when expressed in *ARH1*KO MEFs, the mutant proteins exhibited a wide variation of ARH1 catalytic activity (4–55% of WT activity) (Tables 1 and 2). However, ARH1 activity was not detected in *ARH1*KO MEFs transformed with an *ARH1* frameshift or deletion mutant gene. Thus, some ARH1 proteins encoded by mutant genes isolated from lung adenocarcinoma, had 55% of WT activity when expressed in *ARH1*KO MEFs. Similarly, some tumors that developed from *ARH1* heterozygous MEFs had *ARH1* mutant alleles, which encoded proteins that when expressed in *ARH1*KO MEFs, had 42% of WT activity (Tables 1 and 2). These data suggested that effects on ARH1 enzymatic activity alone were not the sole basis for tumorigenesis. To examine this further, we used *ARH1*KO MEFs stably transformed with *ARH1* mutant and WT genes and assessed the effects of *ARH1* mutations on tumorigenesis.

### Proliferation of *ARH1*KO cells expressing WT and mutant *ARH1* genes from *ARH1*^+/−^ mouse lung adenocarcinoma and *ARH1^+/−^* MEFs injected in nude mice

**Figure 2:**
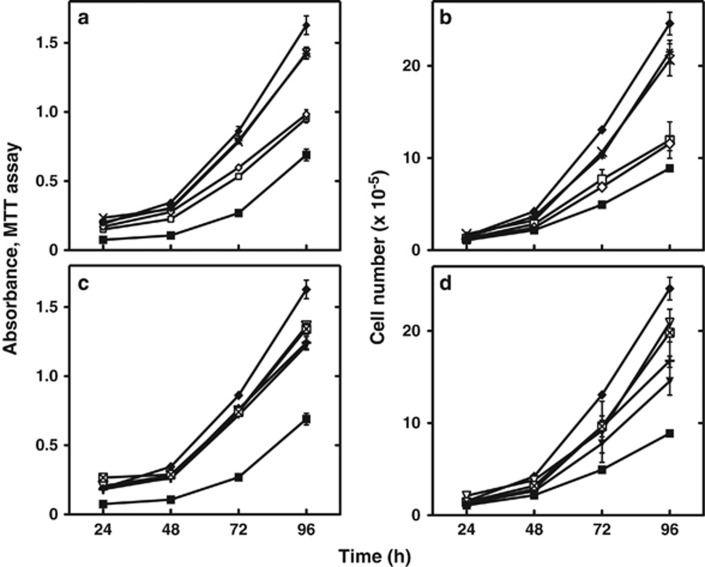
Proliferation of *ARH1*KO cells expressing mutant *ARH1* genes isolated from *ARH1*^*+/-*^ MEFs injected in nude mice. (**a**) *ARH1*KO+WT (▪), *ARH1*KO Mock (♦), *ARH1*KO+mt7 (**×**), *ARH1*KO+mt9 (□), *ARH1*KO+mt13 (◊), *ARH1*KO+mt14 (∗) MEFs (1 × 10^5^) MEFs (5 × 10^3^) were seeded in 96-well plates and MTT assays were performed after growth for the indicated time. (**b**) *ARH1*KO+WT (▪), *ARH1*KO Mock (♦), *ARH1*KO+mt7 (**×**), *ARH1*KO+mt9 (□), *ARH1*KO+mt13 (◊), *ARH1*KO+mt14 (∗) MEFs (1 × 10^5^) were seeded in 100 mm dishes and cell counting was performed after growth for the indicated time. (**c**) *ARH1*KO+WT (▪), *ARH1*KO Mock (♦), *ARH1*KO+mt8 (▾), *ARH1*KO+mt10 (∇), *ARH1*KO+mt11 (+), *ARH1*KO+mt12 (⊠) MEFs (5 × 10^3^) were treated and assayed as in **a**. (**d**) *ARH1*KO+WT (▪), *ARH1*KO Mock (□), *ARH1*KO+mt8 (▾), *ARH1*KO+mt10 (∇), *ARH1*KO+mt11 (+), *ARH1*KO+mt12 (⊠) MEFs (1 × 10^5^) were treated as in **b** before cell counting assays. Data are means±s.e.m. of values from six assays performed in three experiments. (**a**,**b**) Pairwise comparison showed all mutations were significantly different from *ARH1*KO+WT, and *ARH1*KO Mock, +mt7 and +mt14 were significantly different from *ARH1*KO+mt9 and +mt13 (all at *P*<0.001). (**c**,**d**) Pairwise comparison showed all mutations were significantly different from *ARH1*KO+WT and *ARH1*KO mock (all at *P*<0.001).

We reported previously that the proliferation of *ARH1*KO MEFs was faster than that of *ARH1*KO+wt and *ARH1* WT MEFs.^32^ To characterize the *ARH1* mutations, their effects on rates of proliferation of *ARH1*KO *MEFs* transformed with empty vector (*ARH1*KO+mock) were compared with *ARH1*KO MEFs transformed with an *ARH1* WT gene that has 100% ARH1 activity (*ARH1*KO+WT1) and *ARH1*KO MEFs transformed with all *ARH1* mutant genes (4–55% of WT activity, from *ARH1*KO+mt1 to *ARH1*KO+mt14) (Figures 1 and 2). Interestingly, the proliferation rate of *ARH1*KO MEFs transformed with all mutant genes (from *ARH1*KO+mt1 to *ARH1*KO+mt14, 4–55% of WT activity) was significantly (*P*<0.0001) faster than that of *ARH1*KO+WT1 MEFs, and slower (*P*<0.01 to 0.0001) than that of *ARH1*KO Mock MEFs (Figures 1 and 2). A comparison of *ARH1*KO MEFs transformed with all *ARH1* mutant genes showed that the proliferation rate of MEFs transformed with a mutant gene expressing a protein having low ARH1 activity (4-5% of WT activity MEFs *ARH1*KO+mt2 or +mt3) was significantly faster (*P*<0.01) than that of MEFs transformed with mutant genes expressing proteins having high ARH1 activity (42–55% of WT activity, *ARH1*KO+mt1 or +mt6) (Figure 1). Proliferation of *ARH1*KO MEFs containing *ARH1* mutant genes expressing proteins with intermediate ARH1 activity (15–18% of WT activity, *ARH1*KO+mt7 or +mt14) was significantly (*P*<0.01) faster than those containing high ARH1 activity (37–42% of WT activity, *ARH1*KO+mt9 or +mt13) (Figure 2). Thus, in the case of *ARH1* mutations leading to tumor development, the rate of proliferation of transformed *ARH1*KO MEFs depended upon the levels of ARH1 activity. These data also suggested that the proliferation assay and enzymatic activity were not good surrogates for tumorigenesis.

### Effects of *ARH1* mutations of MEFs on growth and colony formation in soft agar

**Figure 3:**
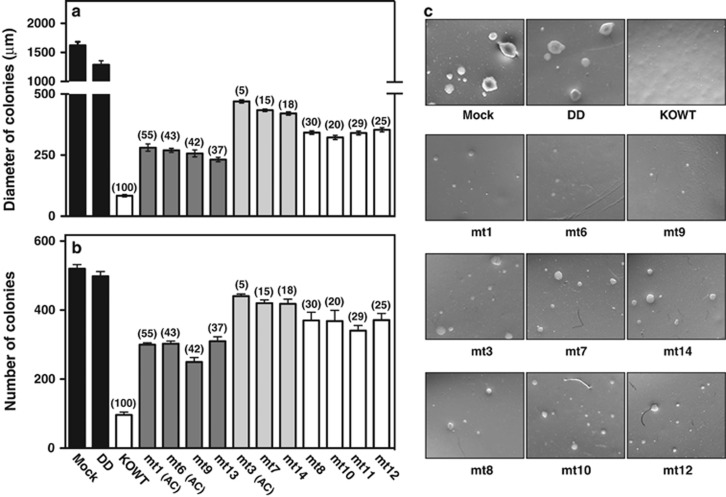
Effects of *ARH1* mutations on growth of MEFs in soft agar. (**a**) Diameters of *ARH1*KO MEFs and +DD (black bars, <1% ARH1 activity of WT) MEFs, *ARH1*KO+WT MEFs (open bar), *ARH1*KO MEFs with mutants that have 36–55% activity of WT (dark-gray bars), *ARH1*KO with mutants that have 5–15% activity of WT (light-gray bars), and *ARH1*KO with mutants that have 20–30% activity of WT (open bars) formed colonies in soft agar after incubation for 42 days (37 ºC, with 5% CO_2_). In all instances, *ARH1* WT genes were isolated to confirm the absence of mutations. Data are means±s.e.m. of values from three experiments with duplicate assays per experiment with each cell line for colony formation in soft agar. Pairwise comparison showed all MEFs containing mutant genes were significantly different from *ARH1*KO+WT, *ARH1*KO mock and +DD MEFs (*P*<0.001). Also, *ARH1*KO+mt1, +mt6, +mt9 and +mt13 (36–55% activity of WT) MEFs were significantly different from *ARH1*KO+mt3, +mt7 *and* +mt14 MEFs (5–15% activity of WT) (*P*<0.05). Numbers in parentheses indicate the % ARH1 activity of *ARH1*KO+WT MEFs. (AC): *ARH1* mutant gene from lung adenocarcinoma in *ARH1*^+/−^ mice. (**b**) Number of *ARH1*KO MEFs and *ARH1*KO+DD MEFs (black bars, <1% ARH1 activity of WT), *ARH1*KO+WT MEFs (open bar), *ARH1*KO MEFs with mutants that have 36–55% activity of WT (dark-gray bars), *ARH1*KO MEFs with mutants that have 5–15% activity of WT (light-gray bars), and *ARH1*KO MEFs with mutants that have 20–30% activity of WT (open bars) that formed colonies in soft agar after incubation for 42 days (37 ºC, with 5% CO_2_). Data are means±s.e.m. of values from three experiments with duplicate assays per experiment with each cell line for colony formation in soft agar. Pairwise comparison showed all MEFs containing mutant genes were significantly different from *ARH1*KO+WT, Mock and +DD MEFs (*P*<0.01). Also, *ARH1*KO+mt1, +mt6, +mt9 and +mt13 MEFs (36–55% activity of WT) were significantly different from *ARH1*KO+mt3, +mt7 *and* +mt14 MEFs (5–15% activity of WT) (*P*<0.05). (**c**) Appearance (x 100) of colonies in soft agar after 32 days of growth of *ARH1*KO MEFs transformed with *ARH1* WT and mutant genes.

Previously, we found that *ARH1*KO MEFs, but not *ARH1* WT and *ARH1*KO+WT MEFs formed colonies in soft agar.^32^ The soft agar colony formation assay is a common method to observe anchorage-independent growth, which correlates with tumorigenesis.^36^ All *ARH1*KO+mt1 to *ARH1*KO+mt14 MEFs transformed with *ARH1* mutant genes and the DD mutant gene formed colonies in soft agar, whereas *ARH1*KO MEFs transformed with the *ARH1* WT1 gene did not (Figure 3). Diameter of colonies with all mutant MEFs (*ARH1*KO+mt1 to +mt14) was significantly smaller than that of colonies formed by *ARH1*KO mock and DD mutant MEFs (Figures 3a and c). Diameters of colonies from MEFs transformed with the low catalytic activity group of ARH1 mutants (*ARH1*KO+mt3, +mt7, and +mt14, 5–15% of WT activity) were larger (*P*<0.01) than that of colonies formed by the high activity group (*ARH1*KO+mt1, +mt6, +mt9, and +mt13, 37–55% of WT activity), but not larger than colonies formed by the intermediate ARH1 activity group (*ARH1*KO+mt8, +mt10, +mt11, and +mt12, 20–30% of WT activity) (Figures 3a and c).

Number of colonies formed by MEFs transformed with any of the mutant MEFs (*ARH1*KO +mt1 to +mt14) was greater (*P*<0.01) than that of colonies seen with *ARH1*KO+WT1 MEFs, but was fewer (*P*<0.01) than those formed by *ARH1*KO mock, and DD MEFs (Figures 3b and c). Data regarding diameter of colonies in soft agar with the different groups of MEFs were similar to data related to the number of colonies. The numbers of colonies seen with the low ARH1 activity MEF group (*ARH1*KO+mt3, mt7, and mt14, 5–15% of WT activity) were greater (*P*<0.05) than those seen with the intermediate ARH1 activity group (*ARH1*KO+mt8, mt10, mt11, and mt12, 20–30% of WT activity) and the high activity group (*ARH1*KO+mt1, +mt6, +mt9, and +mt13, 37–55% of WT activity) (Figures 3b and c). These data indicate that MEFs transformed with *ARH1* mutant genes encoding proteins with residual ARH1 activity display anchorage-independent growth in soft agar with the number of colonies and diameters dependent on hydrolase activity.

### Effects of *ARH1* mutant gene on growth of MEFs in nude mice

**Figure 4:**
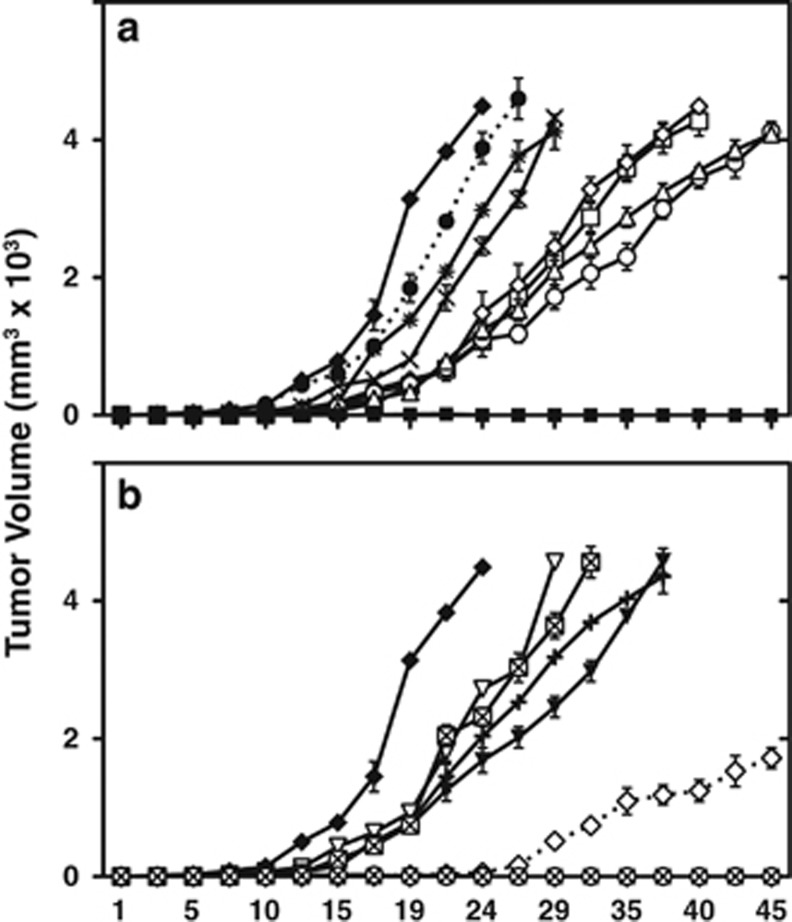
Tumor formation by *ARH1*-deficient MEFs transformed with *ARH1* mutant genes. (**a**) *ARH1*KO+WT1 (▪), *ARH1*KO mock (♦), *ARH1*KO*+*mt1 (○), *ARH1*KO+mt3 (●), *ARH1*KO+mt6 (▵), *ARH1*KO+mt7 (**×**), *ARH1*KO+mt9 (□), *ARH1*KO+mt13 (◊), *ARH1*KO+mt14 (*) MEFs (1 × 10^6^) were injected subcutaneously in nude mice. In all instances, *ARH1* WT genes were isolated to confirm the absence of mutations. Pairwise comparisons indicate that all *ARH1*KO+mutant MEFs were different from *ARH1*KO Mock MEFs and *ARH1*KO+WT1 (*P*<0.001, *P*<0.01). (**b**) *ARH1*KO+WT (▪), *ARH1*KO Mock (♦), *ARH1*KO+mt8 (▾), *ARH1*KO+mt10 (∇), *ARH1*KO+mt11 (+), *ARH1*KO+mt12 (⊠), *ARH1*KO+WT2 (◊), *ARH1*KO+WT6 (⊗) MEFs (1 × 10^6^) were injected subcutaneously in nude mice. Width plus length of tumor masses was measured three times per week thereafter. Data are means±s.e.m. of values from five mice. Experiments were replicated three times (*n*=15). Pairwise comparisons indicate that all *ARH1*KO+mutant MEFs were different from *ARH1*KO Mock MEFs, *ARH1*KO+WT1, +WT2 and +WT6 (*P*<0.001, *P*<0.01). *ARH1*KO+WT6 was different from *ARH1*KO+WT2 (*P*<0.001), but not different from *ARH1*KO+WT1 (*P*>0.05).

Growth of cells in athymic nude mice was used as a measure of tumorigenecity. It was observed previously that *ARH1* genotype affected tumorigenesis; *ARH1*KO and *ARH1* heterozygous MEFs, but not *ARH1* WT and *ARH1*KO+WT MEFs developed tumors in nude mice.^32^ Using *ARH1*KO MEFs transformed with *ARH1* mutant genes, the effects of *ARH1* mutant genes and activities of encoded proteins on subcutaneous tumor mass in athymic nude mice were determined. *ARH1*KO mock MEFs formed tumors in nude mice, whereas *ARH1*KO MEFs transformed with *ARH1* WT1 gene did not (Figure 4). Interestingly, all *ARH1*KO MEFs transformed with *ARH1* mutant genes formed tumors in nude mice (Figure 4). The growth rates of tumors formed in *ARH1*KO MEFs transformed with mutant genes were significantly (*P*<0.001–0.0001) different from *ARH1*KO mock MEFs (Figure 4a). Also, the growth rates of tumors resulting from *ARH1*KO MEFs transformed with *ARH1* mutant genes encoding proteins of the high catalytic activity group (*ARH1*KO+mt1, +mt6, +mt9, and +mt13, 37–55% of WT activity) were significantly (*P*<0.001) slower than those transformed with the low ARH1 activity group (*ARH1*KO+mt3, +mt7 and +mt14, 5–15% of WT activity) (Figure 4a). Growth rates of tumors observed with the intermediate ARH1 activity group (*ARH1*KO+mt8, +mt10, +mt11, and +mt12, 20–30% of WT activity) placed between the low ARH1 activity group (*ARH1*KO+mt3, +mt7 and +mt14, 5–15% of WT activity) and high ARH1 activity group (*ARH1*KO+mt1, +mt6, +mt9, and +mt13, 37–55% of WT activity) (Figure 4b). In addition, *ARH1*KO MEFs transformed with a WT gene but expressing low levels of ARH1 protein and activity (KOWT2, 9% of WT activity) developed tumors, but they grew at a slower rate (*P*<0.01) than *ARH1*KO MEFs transformed with *ARH1* mutant genes having similar ARH1 activity (Figure 4b). Interestingly, growth of tumors seen with *ARH1*KO MEFs transformed with *ARH1* WT gene but expressing intermediate level of ARH1 protein and activity (KOWT6, 43% of WT activity) did not develop tumors in nude mice and were thus similar to *ARH1*KO+WT1 that was designated as 100% ARH1 activity. Thus, all *ARH1*KO MEFs transformed with *ARH1* mutant genes developed tumors, and were thus similar to *ARH1*KO mock MEFs rather than *ARH1*KO+WT MEFs. Further, the levels of expression of the WT and mutant gene were critical to tumor potential.

### *ARH1* gene mutations in human cancer

**Figure 5:**
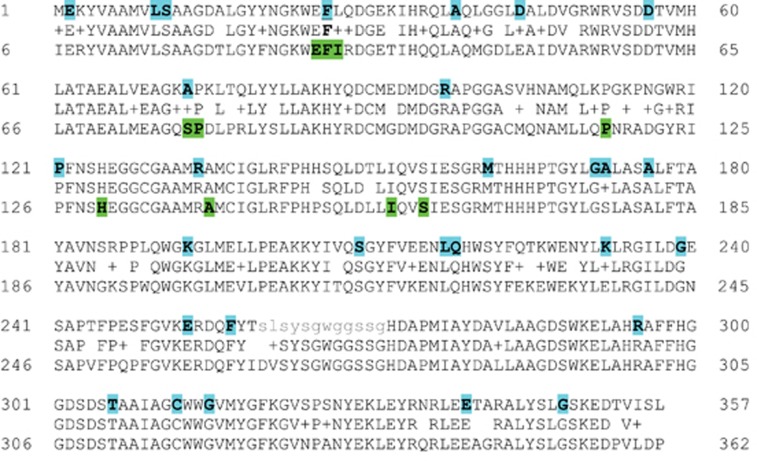
*ARH1* mutation sites in human and murine cancer with ARH1 protein pairwise alignments. Eighty-three percent identities and 91% similarities (middle line) were seen between human *ARH1* (upper line) and murine *ARH1* (lower line) in ARH1 protein alignments. Highlight shows the *ARH1* mutation sites in human and murine cancer.

Based on our tumorigenesis data, it appears that *ARH1* deficiency and mutations were associated with development of lung adenocarcinoma and other cancers. Next, we asked whether human tumors may have *ARH1* mutations and whether the mutations would preferentially occur in exons encoding the catalytic site, as was the case in the murine model. The human cancer database used to search for *ARH1* mutation data was the COSMIC database from Trust Sanger Institute, Genome Research Limited (England). Thirty-two *ARH1* mutations in human cancers (for example, lung, breast and colon) were found in the COSMIC database (Table 3). *ARH1* mutations were observed in human *ARH1* exons 3 and 4, which are equivalent to mouse *ARH1* exons 2 and 3 (Figure 5). The *ARH1* mutations in human cancer were mainly missense mutations with single-base substitution (71.2%, 23 out of 32) similar to the data seen with *ARH1* heterozygous mice (Supplementary Tables 1 and 2). The most frequent mutations of the coding strand were G>T (9 out of 30, 30%), G>A (6 out of 30, 20%) and C>T (6 out of 30, 20%) (Supplementary Table 2). In particular, human *ARH1* gene mutations were more frequent in lung cancer (1.6%) than in cancers of other tissues (Supplementary Table 3). All *ARH1* mutations in human and mouse were seen in parts of the coding region that comprise the active site (Figure 5). Some of the *ARH1* sites mutated in the human gene were similar in location to those found in the mouse *ARH1* gene. Also, the human *ARH1* equivalent amino acid to mouse *ARH1* D61, which was shown previously to be critical for ARH1 activity, is D56. It was found to be mutated in human cancer.

As tumorigenesis was observed in both *ARH1*-deficient and heterozygous mice, ARH1 has properties of a tumor-suppressor gene, and cancers follow a two-hit model.^32^ In agreement, we reported that 6 of 16 lung adenocarcinomas found in *ARH1* heterozygous mice had LOH.^32^ We, therefore, also looked for LOH involving the human *ARH1* gene as a potential mechanism for inactivation of ARH1 in human cancers. *ARH1* LOH in human cancers was found in various types of tumors and tissues (Supplementary Table 4). In the human cancer database, percentage of LOH in lung (15.1%) and kidney (18.0%) cancers was greater than that observed in other tissues. Based on these data, it appears that ARH1 may participate in the pathogenesis of both human, as well as murine cancer.

## Discussion

Previously, we reported that *ARH1* MEF genotype affected cell proliferation, anchorage-independent growth and tumorigenesis in nude mice.^32^ As expected, the transformed *ARH1* WT gene rescued the phenotypes of the *ARH1*-deficient MEF *in vitro* and *in vivo*.^32^ *ARH1* heterozygous mice and MEFs injected into nude mice developed tumors, which appeared to be the result of loss of the functioning *ARH1* alleles, either by LOH or mutation.

In this report, we looked at the effects of *ARH1* gene mutations on ARH1 activity *in vitro*, and on their effects on the cell biological properties of *ARH1*-deficient MEFs. We found 12 missense mutations and 2 deletion mutations in the murine *ARH1* gene. Surprisingly, a wide range of ARH1 activities were observed (4–55% of WT activity) in *ARH1*KO MEFs transformed with *ARH1* mutant genes. As ARH1 activity was measured with an ADP-ribosyl(arginine) substrate, the differential ARH1 activities of the WT and mutated ARH1 proteins are relative to catalytic activity with ADP-ribosyl(arginine).^30, 33^ When the WT and mutated ARH1 proteins use ADP-ribosylated proteins as substrates as would occur *in vivo*, the relative activities would reflect the context of the ADP-ribose-arginine in the protein quaternary structure. ADP-ribosylated protein substrates formed under physiological conditions by ARTs may prove better or worse than the model substrate for WT *ARH1*. Similarly, *ARH1* mutants may have more or less activity toward the *in vivo* substrates than WT *ARH1*.

Furthermore, even levels of WT hydrolase were critical to tumorigenesis. *ARH1*KO MEFs transformed with WT2 (9% of WT activity) (KO+WT2) formed tumors in nude mice. However, *ARH1*KO MEFs transformed with WT6 (43% of WT activity) (KO+WT6) and *ARH1*KO MEFs transformed WT1 (100% of WT activity) did not form tumors in nude mice, whereas *ARH1*KO MEFs transformed with an *ARH1* mutant with activity similar to KO+WT6 resulted in tumors in nude mice. The data suggest that the mutated ARH1 proteins may also be unable to hydrolyze *in vivo* ADP-ribosylated protein found in the tumor, thus resulting in cancers, despite the presence of ARH1 enzymatic activity. Thus, *ARH1* mutation or level of ARH1 activity may affect rate of cell proliferation and tumorigenesis in nude mice.

Mono-ADP-ribosylation of proteins (that is, Gαs, Ras, ExoS, P2X7 and HNP-1) on arginine residues by mammalian ARTs or bacterial ADP-ribosylating toxins affected function by different mechanisms.^15, 24, 25, 37, 38, 39, 40^ The resulting effects on acceptor proteins can be activating or inactivating.^41^ ART1, an arginine-specific ART expressed on the surface of airway epithelial cells and neutrophils,^42, 43^ catalyzes the transfer of the ADP-ribose moiety of NAD to arginines (R14, R24) of HNP-1, inhibiting its antibacterial and cytotoxic activities.^24, 40^ Cholera toxin ADP-ribosylation inhibits Gαs intrinsic GTPase activity, which converts the active GTP-bound Gαs to the inactive, GDP-bound Gαs. ADP-ribosylation of arginine (R187) by inhibiting GTPase activity, prolongs the lifetime of the active, GTP-bound Gαs. The resulting persistent activation of adenylyl cyclase by Gαs-GTP increases cyclic AMP content.^15^ Moreover, *Pseudomonas aeruginosa* exoenzyme S (ExoS) catalyzes the ADP-ribosylation of Ras at Arg41 and Arg128.^37^ ADP-ribosylated of Arg41 inhibits the binding of the guanine nucleotide exchange factor, which inhibits downstream effector (Raf) signaling.^44^

Our finding with murine tumors may be applicable to human cancers. Some human tumors have *ARH1* mutations encoding amino acids similar in position to those seen with *ARH1* genes isolated from murine tumors. Based on the human somatic tumor mutation database and our results of murine *ARH1* gene mutations, ARH1 appears to be tumor-suppressor gene.

## Materials and methods

Materials and methods for cell culture, quantification of viable cells, isolation of proteins from cultured cells and assessing colony formation in soft agar, tumorigenicity in nude mice and cholera toxin activity *in vitro* and *in vivo* were described previously.^31, 32^

### COSMIC database search for *ARH1* gene

Data in Table 3, Supplementary Table 1, 3 and 4 were based on the COSMIC database <http://cancer.sanger.ac.uk/cancergenome/projects/cosmic/> (COSMIC v67, v68 and v69 release). References obtained from the cosmic database are available in the supplementary section (1-11).

### Transfection and clonal selection

*ARH1* WT (coding region), double-mutant (D60A, D61A), and all tumor-derived mutant (mt1-14) cDNA were inserted into pcDNA 3.1 (Invitrogen, Life technology, Carlsbad, CA, USA) vectors carrying a Zeocin resistance marker. *ARH1*-deficient (KO) MEFs, passage 5, were transfected using Lipofectamine and Plus reagent (Invitrogen, Life technology) with pcDNA 3.1, pcDNA 3.1+WT (+WT), pcDNA 3.1 +double-mutant (DD), and pcDNA 3.1+mutant 1–14 (mt1-14) vectors. Transfected cells were grown for 2–3 weeks with Zeocin (1 mg/ml), at which times individual colonies with resistance to Zeocin were selected and grown in 96-well plates. Expression of ARH1 WT, the DD double-mutant and all murine tumor mutants was confirmed with western blot analyses. *ARH1*KO MEFs transformed with control vector were designated *ARH1*KO mock, those with *ARH1* WT cDNA were designated *ARH1*KO+WT, those with *ARH1* double-mutant as *ARH1*KO+DD and those with other mutations as *ARH1*KO+mt1-14 MEFs.

### Statistics

Two-way analysis of variance (repeated-measures analysis of variance methods using Bonferroni's and Tukey's multiple-comparison tests) were used to evaluate the effect of *ARH1* gene mutations on the proliferation of MEFs and their tumorigenic potential. To minimize false-positive conclusions, all pairwise mutation comparisons were performed only if the overall model performance was significant. These comparisons were adjusted for multiple testing. A Kruskal–Wallis test was used to compare the means from each experiment and the distribution of mutations in each experiment. Tukey's multiple-comparison tests were used to compare the diameter and number of colonies in soft agar for pairwise comparisons of *ARH1* gene mutations. Maximum tumor volume was used to compare *ARH1* gene mutations. Finally, a logistic regression was used with binary outcome variables. All statistical tests were considered significant at the 0.05 level.

## Figures and Tables

**Table 1:** *ARH1* mutations in lung adenocarcinoma from *ARH1* heterozygous female mice

*Mouse ARH1 mutation*	*Amino-acid mutation*	*CDS^c^ mutation*	*Sample number*^a^	*Mutation type*	ARH1 *activity (%)*
mt1	p.I32V	c.94A>G	2	Substitution – missense	55.3±1.2
mt2	p.F31S	c.92T>C	2	Substitution – missense	4±1.1
mt3	p.F31S, p.I32V	c.92T>C, c.94A>G	1	Substitution – missense	4.9±1.3
mt4	p.S78S, P79P stop codon	c.237G>del^b^	1	Deletion – frameshift	None detected
mt5	p.S78S, P79R stop codon	c.233C>del^b^	1	Deletion – frameshift	None detected
mt6	p.P117L	c.350C>T	3	Substitution – missense	42.9±1.4

Abbreviations: ARH, ADP-ribose-acceptor hydrolase; MEFs, mouse embryonic fibroblasts.

All mutations were in exon 2.

a Sample number: number of samples with a *ARH1* gene mutation.

b Deletion mutant, frameshift: no recombinant protein, no stably *ARH1*-deficient MEFs.

c CDS: coding DNA sequence.

**Table 2:** *ARH1* mutations in tumors of female nude mice injected subcutaneously with *ARH1* heterozygous MEFs

*Mouse ARH1 mutation*	*Amino-acid mutation*	*CDS^c^ mutation*	*Exon*^a^	*Sample number*^b^	ARH1 *activity (%)*
mt7	p.E30G, p.F31S	c.89A>G, c.92T>C	2	1	14.7±0.5
mt8	p.H130P	c.389A>C	3	2	29.5±1.0
mt9	p.H130L	c.389A>T	3	2	42.2±0.6
mt10	p.A140T	c.408G>A	3	2	20.5±0.4
mt11	p.L145R, p.R146G	c.434T>G, c.435G>C, c.436A>G	3	1	28.4±0.6
mt12	p.I157T	c.470T>C	3	3	24.8±0.5
mt13	p.S160C	c.478A>T	3	2	36.7±1.2
mt14	p.F31S	c.92T>C, c93C>G	2	2	14.8±1.9

Abbreviations: ARH, ADP-ribose-acceptor hydrolase; cDNA, complementary DNA; MEFs, mouse embryonic fibroblasts.

Histology: fibrosarcoma in all subcutaneous tumors of female nude mice.

Mutation type: substitution – missense.

a Exon number of *ARH1* cDNA gene.

b Sample number: number of samples with a *ARH1* gene mutation.

c CDS: coding DNA sequence.

**Table 3:** *ARH1* gene mutations reported in human cancer^a^

*No.*	*CDS mutation*	*AA mutation*	*Primary tissue*	*Histology*	*Count*	*Zygosity*	*Gender*	*Type*
1	c.4G>T	p.E2	Lung	AC^2^^a^	1	Unknown	Male	Substitution – nonsense
2	c.30G>T	p.L10L	Lung	SCC^3^^a^	1	Unknown	Female	Substitution – coding silent
3	c.31 A>C	p.S11R	Lung	SCC	1	Unknown	Female	Substitution – missense
4	c.78C>T	p.F26F	Lung	SCC	1	Unknown	Male	Substitution – coding silent
5	c.113C>T	p.A38V	Kidney	Renal cell carcinoma	1	Unknown	Male	Substitution – missense
6	c.140 A>T	p.D47V	Breast	Carcinoma	1	Heterozygous	Female	Substitution – missense
7	c.166G>A	p.D56N	Stomach, intestinal	AC	1	Unknown	Male	Substitution – missense
8	c.218C>A	p.A73D	Breast	Carcinoma	1	Heterozygous	Female	Substitution – missense
9	c.289C>T	p.R97W	Colon	AC	1	Heterozygous	Female	Substitution – missense
10	c.362C>T	p.P121L	Lung	SCC	1	Unknown	Unknown	Substitution – missense
11	c.400C>T	p.R134W	Lung	SCC	1	Unknown	Male	Substitution – missense
12	c.401G>A	p.R134Q	Prostate	AC	1	Unknown	Unknown	Substitution – missense
13	c.438C>T	p.S146S	Cecum	AC	1	Heterozygous	Female	Substitution – coding silent
14	c.481 A>G	p.M161V	Lung	AC	1	Unknown	Male	Substitution – missense
15	c.513G>A	p.G171G	Urinary tract, bladder	Carcinoma	1	Unknown	Male	Substitution – coding silent
16	c.510delG	p.A172fs 12	Colon	AC	1	Heterozygous	Female	Deletion – frameshift
17	c.526G>T	p.A176S	Lung	AC	1	Unknown	Female	Substitution – missense
18	c.577 A>T	p.K193	Endometrium	Carcinoma	1	Heterozygous	Female	Substitution – nonsense
19	c.625 T>G	p.S209A	Endometrium	Carcinoma	1	Heterozygous	Female	Substitution – missense
20	c.649C>G	p.L217V	Lung	AC	1	Unknown	Female	Substitution – missense
21	c.653 A>C	p.Q218P	Ovary	Serous carcinoma	1	Heterozygous	Female	Substitution – missense
22	c.688_689insACCTA	p.K232fs 2	Esophagus	AC	1	Unknown	Female	Insertion – frameshift
23	c.716G>T	p.G239V	Lung	AC	1	Unknown	Male	Substitution – missense
24	c.757G>T	p.E253	Lung	SCC	1	Unknown	Male	Substitution – nonsense
25	c.771C>A	p.F257L	Endometrium	Carcinoma	1	Heterozygous	Female	Substitution – missense
26	c.884G>A	p.R295Q	Endometrium	Carcinoma	1	Heterozygous	Female	Substitution – missense
27	c.884G>T	p.R295L	Lung	AC	2	Unknown	Male	Substitution – missense
28	c.916 A>G	p.T306A	Lung	SCC	1	Unknown	Male	Substitution – missense
29	c.936C>A	p.C312	Breast	Carcinoma	1	Heterozygous	Female	Substitution – nonsense
30	c.944G>T	p.G315V	Colon	AC	1	Heterozygous	Male	Substitution – missense
31	c.1017G>A	p.E339E	Lung	AC	1	Unknown	Male	Substitution – coding silent
32	c.1043G>A	p.G348E	Breast	AC	1	Heterozygous	Female	Substitution – missense

Abbreviations: AC^2a^, adenocarcinoma; SCC^3a^, squamous cell carcinoma.

a These data were obtained from the COSMIC database (COSMIC v67, v68 and v69 release) http://cancer.sanger.ac.uk/cancergenome/projects/cosmic/.

## Supplementary Material

Supplementary References

## References

[bib1] 1Cancer Genome Atlas Research N Comprehensive molecular profiling of lung adenocarcinomaNature 2014; 511: 543–550. 25079552 10.1038/nature13385 PMC4231481

[bib2] 2Freed-Pastor WA, Prives C. Mutant p53: one name, many proteins. Genes Dev 2012; 26: 1268–1286. 22713868 10.1101/gad.190678.112 PMC3387655

[bib3] 3Lania A, Mantovani G, Spada A. G protein mutations in endocrine diseases. Eur J Endocrinol 2001; 145: 543–559. 11720871 10.1530/eje.0.1450543

[bib4] 4Mao C, Liao RY, Chen Q. BRAF mutation predicts resistance to anti-EGFR monoclonal antibodies in wild-type KRAS metastatic colorectal cancer. J Cancer Res Clin Oncol 2010; 136: 1293–1294. 20514492 10.1007/s00432-010-0922-8 PMC11827788

[bib5] 5Takahashi T, Nau MM, Chiba I, Birrer MJ, Rosenberg RK, Vinocour M et al. p53: a frequent target for genetic abnormalities in lung cancer. Science 1989; 246: 491–494. 2554494 10.1126/science.2554494

[bib6] 6Bode AM, Dong Z. Post-translational modification of p53 in tumorigenesis. Nat Rev Cancer 2004; 4: 793–805. 15510160 10.1038/nrc1455

[bib7] 7Karnoub AE, Weinberg RA. Ras oncogenes: split personalities. Nat Rev Mol Cell Biol 2008; 9: 517–531. 18568040 10.1038/nrm2438 PMC3915522

[bib8] 8Myatt SS, Lam EW. The emerging roles of forkhead box (Fox) proteins in cancer. Nat Rev Cancer 2007; 7: 847–859. 17943136 10.1038/nrc2223

[bib9] 9Hassa PO, Haenni SS, Elser M, Hottiger MO. Nuclear ADP-ribosylation reactions in mammalian cells: where are we today and where are we going? Microbiol Mol Biol Rev 2006; 70: 789–829. 16959969 10.1128/MMBR.00040-05 PMC1594587

[bib10] 10Hsiao SJ, Smith S. Tankyrase function at telomeres, spindle poles, and beyond. Biochimie 2008; 90: 83–92. 17825467 10.1016/j.biochi.2007.07.012

[bib11] 11Schreiber V, Dantzer F, Ame JC, de Murcia G. Poly(ADP-ribose): novel functions for an old molecule. Nat Rev Mol Cell Biol 2006; 7: 517–528. 16829982 10.1038/nrm1963

[bib12] 12Jungmichel S, Rosenthal F, Altmeyer M, Lukas J, Hottiger MO, Nielsen ML. Proteome-wide identification of poly(ADP-Ribosyl)ation targets in different genotoxic stress responses. Mol Cell 2013; 52: 272–285. 24055347 10.1016/j.molcel.2013.08.026

[bib13] 13Moss J, Vaughan M. ADP-Ribosylating Toxins and G Proteins: Insights into Signal Transduction. American Society for Microbiology: , Washington, DC, 1990. 10.1126/science.250.4982.841-a 17759980

[bib14] 14Corda D, Di Girolamo M. Functional aspects of protein mono-ADP-ribosylation. EMBO J 2003; 22: 1953–1958. 12727863 10.1093/emboj/cdg209 PMC156081

[bib15] 15Cassel D, Pfeuffer T. Mechanism of cholera toxin action: covalent modification of the guanyl nucleotide-binding protein of the adenylate cyclase system. Proc Natl Acad Sci USA 1978; 75: 2669–2673. 208069 10.1073/pnas.75.6.2669 PMC392624

[bib16] 16Margarit SM, Davidson W, Frego L, Stebbins CE. A steric antagonism of actin polymerization by a salmonella virulence protein. Structure 2006; 14: 1219–1229. 16905096 10.1016/j.str.2006.05.022

[bib17] 17Tezcan-Merdol D, Nyman T, Lindberg U, Haag F, Koch-Nolte F, Rhen M. Actin is ADP-ribosylated by the Salmonella enterica virulence-associated protein SpvB. Mol Microbiol 2001; 39: 606–619. 11169102 10.1046/j.1365-2958.2001.02258.x

[bib18] 18Berti PJ, Blanke SR, Schramm VL. Transition state structure for the hydrolysis of NAD catalyzed by diphtheria toxin. J Am Chem Soc 1997; 119: 12079–12088. 19079637 10.1021/ja971317a PMC2601651

[bib19] 19Koch-Nolte F, Kernstock S, Mueller-Dieckmann C, Weiss MS, Haag F. Mammalian ADP-ribosyltransferases and ADP-ribosylhydrolases. Front Biosci 2008; 13: 6716–6729. 18508690 10.2741/3184

[bib20] 20Moss J, Jacobson MK, Stanley SJ. Reversibility of arginine-specific mono(ADP-ribosyl)ation: identification in erythrocytes of an ADP-ribose-L-arginine cleavage enzyme. Proc Natl Acad Sci USA 1985; 82: 5603–5607. 2994036 10.1073/pnas.82.17.5603 PMC390599

[bib21] 21Moss J, Oppenheimer NJ, West RE Jr., Stanley SJ. Amino acid specific ADP-ribosylation: substrate specificity of an ADP-ribosylarginine hydrolase from turkey erythrocytes. Biochemistry 1986; 25: 5408–5414. 3778868 10.1021/bi00367a010

[bib22] 22Tsuge H, Nagahama M, Oda M, Iwamoto S, Utsunomiya H, Marquez VE et al. Structural basis of actin recognition and arginine ADP-ribosylation by Clostridium perfringens iota-toxin. Proc Natl Acad Sci U S A 2008; 105: 7399–7404. 18490658 10.1073/pnas.0801215105 PMC2387182

[bib23] 23Moss J, Stanley SJ, Nightingale MS, Murtagh JJ Jr., Monaco L, Mishima K et al. Molecular and immunological characterization of ADP-ribosylarginine hydrolases. J Biol Chem 1992; 267: 10481–10488. 1375222

[bib24] 24Paone G, Wada A, Stevens LA, Matin A, Hirayama T, Levine RL et al. ADP ribosylation of human neutrophil peptide-1 regulates its biological properties. Proc Natl Acad Sci USA 2002; 99: 8231–8235. 12060767 10.1073/pnas.122238899 PMC123050

[bib25] 25Paone G, Stevens LA, Levine RL, Bourgeois C, Steagall WK, Gochuico BR et al. ADP-ribosyltransferase-specific modification of human neutrophil peptide-1. J Biol Chem 2006; 281: 17054–17060. 16627471 10.1074/jbc.M603042200

[bib26] 26Seman M, Adriouch S, Scheuplein F, Krebs C, Freese D, Glowacki G et al. NAD-induced T cell death: ADP-ribosylation of cell surface proteins by ART2 activates the cytolytic P2X7 purinoceptor. Immunity 2003; 19: 571–582. 14563321 10.1016/s1074-7613(03)00266-8

[bib27] 27Schwarz N, Fliegert R, Adriouch S, Seman M, Guse AH, Haag F et al. Activation of the P2X7 ion channel by soluble and covalently bound ligands. Purinergic Signal 2009; 5: 139–149. 19255877 10.1007/s11302-009-9135-5 PMC2686825

[bib28] 28Moss J, Tsai SC, Adamik R, Chen HC, Stanley SJ. Purification and characterization of ADP-ribosylarginine hydrolase from turkey erythrocytes. Biochemistry 1988; 27: 5819–5823. 3179279 10.1021/bi00415a063

[bib29] 29Smith KP, Benjamin RC, Moss J, Jacobson MK. Identification of enzymatic activities which process protein bound mono(ADP-ribose). Biochem Biophys Res Commun 1985; 126: 136–142. 2982365 10.1016/0006-291x(85)90582-0

[bib30] 30Takada T, Okazaki IJ, Moss J. ADP-ribosylarginine hydrolases. Mol Cell Biochem 1994; 138: 119–122. 7898453 10.1007/BF00928452

[bib31] 31Kato J, Zhu J, Liu C, Moss J. Enhanced sensitivity to cholera toxin in ADP-ribosylarginine hydrolase-deficient mice. Mol Cell Biol 2007; 27: 5534–5543. 17526733 10.1128/MCB.00302-07 PMC1952103

[bib32] 32Kato J, Zhu J, Liu C, Stylianou M, Hoffmann V, Lizak MJ et al. ADP-ribosylarginine hydrolase regulates cell proliferation and tumorigenesis. Cancer Res 2011; 71: 5327–5335. 21697277 10.1158/0008-5472.CAN-10-0733 PMC3399181

[bib33] 33Konczalik P, Moss J. Identification of critical, conserved vicinal aspartate residues in mammalian and bacterial ADP-ribosylarginine hydrolases. J Biol Chem 1999; 274: 16736–16740. 10358013 10.1074/jbc.274.24.16736

[bib34] 34COSMIC (2014) Catalogue of Somatic Mutations in Cancer, 4 February 2014 edn.

[bib35] 35Forbes SA, Bhamra G, Bamford S, Dawson E, Kok C, Clements J et al. The catalogue of somatic mutations in cancer (COSMIC). Curr Protocols Hum Genet/editorial board, Jonathan L Haines [et al] 2008; Chapter 10: Unit 10 11. 10.1002/0471142905.hg1011s57 PMC2705836 18428421

[bib36] 36Dodson MG, Slota J, Lange C, Major E. Distinction of the phenotypes of in vitro anchorage-independent soft-agar growth and in vivo tumorigenicity in the nude mouse. Cancer Res 1981; 41: 1441–1446. 7011536

[bib37] 37Ganesan AK, Frank DW, Misra RP, Schmidt G, Barbieri JT. Pseudomonas aeruginosa exoenzyme S ADP-ribosylates Ras at multiple sites. J Biol Chem 1998; 273: 7332–7337. 9516428 10.1074/jbc.273.13.7332

[bib38] 38Riese MJ, Goehring UM, Ehrmantraut ME, Moss J, Barbieri JT, Aktories K et al. Auto-ADP-ribosylation of Pseudomonas aeruginosa ExoS. J Biol Chem 2002; 277: 12082–12088. 11821389 10.1074/jbc.M109039200

[bib39] 39Simon NC, Aktories K, Barbieri JT. Novel bacterial ADP-ribosylating toxins: structure and function. Nat Rev Microbiol 2014; 12: 599–611. 25023120 10.1038/nrmicro3310 PMC5846498

[bib40] 40Stevens LA, Levine RL, Gochuico BR, Moss J. ADP-ribosylation of human defensin HNP-1 results in the replacement of the modified arginine with the noncoded amino acid ornithine. Proc Natl Acad Sci USA 2009; 106: 19796–19800. 19897717 10.1073/pnas.0910633106 PMC2785246

[bib41] 41Laing S, Unger M, Koch-Nolte F, Haag F. ADP-ribosylation of arginine. Amino Acids 2011; 41: 257–269. 20652610 10.1007/s00726-010-0676-2 PMC3102197

[bib42] 42Balducci E, Horiba K, Usuki J, Park M, Ferrans VJ, Moss J. Selective expression of RT6 superfamily in human bronchial epithelial cells. Am J Respir Cell Mol Biol 1999; 21: 337–346. 10460751 10.1165/ajrcmb.21.3.3638

[bib43] 43Saxty BA, Kefalas P, Yadollahi-Farsani M, MacDermot J. Arginine-specific ADP-ribosyltransferases in leukocytes. J Leukoc Biol 1998; 63: 15–21. 9469468 10.1002/jlb.63.1.15

[bib44] 44Ganesan AK, Vincent TS, Olson JC, Barbieri JT. Pseudomonas aeruginosa exoenzyme S disrupts Ras-mediated signal transduction by inhibiting guanine nucleotide exchange factor-catalyzed nucleotide exchange. J Biol Chem 1999; 274: 21823–21829. 10419499 10.1074/jbc.274.31.21823

