# Supplementary material for: Mutations of the functional ARH1 allele in tumors from ARH1 heterozygous mice and cells affect ARH1 catalytic activity, cell proliferation and tumorigenesis
Source: Oncogenesis. 2015 Jun 1;4(6):e151–. doi: 10.1038/oncsis.2015.5 (PMC4753525; doi:10.1038/oncsis.2015.5)
Supplement: Supplementary References [file oncsis20155x1.docx]

Supplementary references

1. Cancer Genome Atlas Research N. Integrated genomic analyses of ovarian carcinoma. Nature 2011;**474**(7353):609-15.

2. Dulak AM, Stojanov P, Peng S, Lawrence MS, Fox C, Stewart C, et al. Exome and whole-genome sequencing of esophageal adenocarcinoma identifies recurrent driver events and mutational complexity. Nat Genet 2013;**45**(5):478-86.

3. Imielinski M, Berger AH, Hammerman PS, Hernandez B, Pugh TJ, Hodis E, et al. Mapping the hallmarks of lung adenocarcinoma with massively parallel sequencing. Cell 2012;**150**(6):1107-20.

4. Jones S, Zhang X, Parsons DW, Lin JC, Leary RJ, Angenendt P, et al. Core signaling pathways in human pancreatic cancers revealed by global genomic analyses. Science 2008;**321**(5897):1801-6.

5. Lindberg J, Mills IG, Klevebring D, Liu W, Neiman M, Xu J, et al. The mitochondrial and autosomal mutation landscapes of prostate cancer. Eur Urol 2013;**63**(4):702-8.

6. Parsons DW, Jones S, Zhang X, Lin JC, Leary RJ, Angenendt P, et al. An integrated genomic analysis of human glioblastoma multiforme. Science 2008;**321**(5897):1807-12.

7. Parsons DW, Li M, Zhang X, Jones S, Leary RJ, Lin JC, et al. The genetic landscape of the childhood cancer medulloblastoma. Science 2011;**331**(6016):435-9.

8. Pickering CR, Zhang J, Yoo SY, Bengtsson L, Moorthy S, Neskey DM, et al. Integrative genomic characterization of oral squamous cell carcinoma identifies frequent somatic drivers. Cancer Discov 2013;**3**(7):770-81.

9. Rudin CM, Durinck S, Stawiski EW, Poirier JT, Modrusan Z, Shames DS, et al. Comprehensive genomic analysis identifies SOX2 as a frequently amplified gene in small-cell lung cancer. Nat Genet 2012;**44**(10):1111-6.

10. Sjoblom T, Jones S, Wood LD, Parsons DW, Lin J, Barber TD, et al. The consensus coding sequences of human breast and colorectal cancers. Science 2006;**314**(5797):268-74.

11. Zang ZJ, Cutcutache I, Poon SL, Zhang SL, McPherson JR, Tao J, et al. Exome sequencing of gastric adenocarcinoma identifies recurrent somatic mutations in cell adhesion and chromatin remodeling genes. Nat Genet 2012;**44**(5):570-4.
