# Supplementary material for: Mutations of the functional ARH1 allele in tumors from ARH1 heterozygous mice and cells affect ARH1 catalytic activity, cell proliferation and tumorigenesis
Source: Oncogenesis. 2015 Jun 1;4(6):e151–. doi: 10.1038/oncsis.2015.5 (PMC4753525; doi:10.1038/oncsis.2015.5)
Supplement: Supplementary Table 1 [file oncsis20155x2.docx]

**Supplementary Table 1.**

**Distribution of *ARH1* Mutation Type in *ARH1* Heterozygous Mice and MEFs**

| **Mutation Type** | **Mutant samples** | **Percentage** |
| --- | --- | --- |
| Substitution nonsense | 0 | 0 |
| Substitution missense | 12 | 85.7 |
| Substitution synonymous | 0 | 0 |
| Insertion in-frame | 0 | 0 |
| Insertion frame-shift | 0 | 0 |
| Deletion in-frame | 0 | 0 |
| Deletion frame-shift | 2 | 14.3 |
| Complex | 0 | 0 |
| Other | 0 | 0 |
| Total | 14 | 100 |
|  |  |  |
| **Mutation Type** | **Mutant samples** | **Percentage** |
| A>C | 1 | 5.88 |
| A>G | 5 | 29.41 |
| A>T | 2 | 11.76 |
| C>A | 0 | 0.00 |
| C>T | 1 | 5.88 |
| C>G | 1 | 5.88 |
| G>A | 1 | 5.88 |
| G>C | 1 | 5.88 |
| G>T | 0 | 0.00 |
| T>A | 0 | 0.00 |
| T>C | 4 | 23.53 |
| T>G | 1 | 5.88 |
| Total | 17 | 100 |
