# Supplementary material for: Mutations of the functional ARH1 allele in tumors from ARH1 heterozygous mice and cells affect ARH1 catalytic activity, cell proliferation and tumorigenesis
Source: Oncogenesis. 2015 Jun 1;4(6):e151–. doi: 10.1038/oncsis.2015.5 (PMC4753525; doi:10.1038/oncsis.2015.5)
Supplement: Supplementary Table 2 [file oncsis20155x3.docx]

**Supplementary Table 2.**

**Distribution of *ARH1* Mutation Types Reported in Human Cancer** *

| **Mutation Type** | **Mutant samples** | **Percentage** |
| --- | --- | --- |
| Substitution nonsense | 4 | 12.5 |
| Substitution missense | 23 | 71.88 |
| Substitution synonymous | 5 | 15.62 |
| Insertion inframe | 0 | 0 |
| Insertion frameshift | 1 | 3.12 |
| Deletion inframe | 0 | 0 |
| Deletion frameshift | 1 | 3.12 |
| Complex | 0 | 0 |
| Other | 0 | 0 |
| Total | 32 | 100 |
|  |  |  |
| **Mutation Type** | **Mutant samples** | **Percentage** |
| A>C | 2 | 6.67 |
| A>G | 2 | 6.67 |
| A>T | 2 | 6.67 |
| C>A | 3 | 10 |
| C>T | 6 | 20 |
| C>G | 1 | 3.33 |
| G>A | 6 | 20 |
| G>C | 0 | 0 |
| G>T | 9 | 30 |
| T>A | 0 | 0 |
| T>C | 0 | 0 |
| T>G | 1 | 3.33 |
| Total | 30 | 100 |

* These data were searched from COSMIC database (COSMIC v 67 release to v69 release) http://cancer.sanger.ac.uk/cancergenome/projects/cosmic/.
