# Supplementary material for: Mutations of the functional ARH1 allele in tumors from ARH1 heterozygous mice and cells affect ARH1 catalytic activity, cell proliferation and tumorigenesis
Source: Oncogenesis. 2015 Jun 1;4(6):e151–. doi: 10.1038/oncsis.2015.5 (PMC4753525; doi:10.1038/oncsis.2015.5)
Supplement: Supplementary Table 3 [file oncsis20155x4.docx]

**Supplementary Table 3.**

**Tissue Distribution of *ARH1* Gene Mutations Reported in Human Cancer ***

| Tissue | Samples tested | Mutated samples | % mutated |
| --- | --- | --- | --- |
| Adrenal gland | 23 | 0 | 0 |
| Autonomic ganglia | 362 | 0 | 0 |
| Biliary tract | 11 | 0 | 0 |
| Bone | 75 | 0 | 0 |
| Breast | 978 | 4 | 0.4 |
| Central nervous system | 811 | 0 | 0 |
| Cervix | 14 | 0 | 0 |
| Endometrium | 271 | 3 | 1.1 |
| Eye | 34 | 0 | 0 |
| Haematopoietic and lymphoid | 1103 | 0 | 0 |
| Kidney | 611 | 1 | 0.2 |
| Large intestine | 664 | 4 | 0.6 |
| Liver | 827 | 0 | 0 |
| Lung | 861 | 14 | 1.6 |
| Meninges | 55 | 0 | 0 |
| NS | 213 | 0 | 0 |
| Oesophagus | 173 | 1 | 0.6 |
| Ovary | 504 | 1 | 0.2 |
| Pancreas | 691 | 1 | 0.1 |
| Parathyroid | 16 | 0 | 0 |
| Prostate | 424 | 1 | 0.2 |
| Salivary gland | 49 | 0 | 0 |
| Skin | 321 | 0 | 0 |
| Small intestine | 42 | 0 | 0 |
| Soft tissue | 16 | 0 | 0 |
| Stomach | 47 | 1 | 2.1 |
| Thyroid | 17 | 0 | 0 |
| Upper aerodigestive tract | 166 | 0 | 0 |
| Urinary tract | 103 | 1 | 1.0 |
| Total | 9482 | 32 | 0.3 |

* These data were searched from COSMIC database (COSMIC v 67 release to v69 release) http://cancer.sanger.ac.uk/cancergenome/projects/cosmic/.
