# Supplementary material for: Mutations of the functional ARH1 allele in tumors from ARH1 heterozygous mice and cells affect ARH1 catalytic activity, cell proliferation and tumorigenesis
Source: Oncogenesis. 2015 Jun 1;4(6):e151–. doi: 10.1038/oncsis.2015.5 (PMC4753525; doi:10.1038/oncsis.2015.5)
Supplement: Supplementary Table 4 [file oncsis20155x5.docx]

**Supplementary Table 4.**

**Loss of Heterozygosity (LOH) of *ARH1* Gene Observed in Human Cancer ***

| Tissue | Number of samples analyzed | Number of samples with LOH | LOH (%) |
| --- | --- | --- | --- |
| Breast | 852 | 73 | 8.6 |
| Central nervous system | 140 | 8 | 5.7 |
| Endometrium | 246 | 6 | 2.4 |
| Hematopoietic and lymphoid | 192 | 1 | 0.5 |
| Kidney | 300 | 54 | 18.0 |
| Large intestine | 486 | 12 | 2.5 |
| Lung | 476 | 72 | 15.1 |
| Ovary | 462 | 20 | 4.3 |
| Tissue not identified | 30 | 2 | 6.6 |

* These data were searched from COSMIC database (COSMIC v67, v68, and v69 release) http://cancer.sanger.ac.uk/cancergenome/projects/cosmic/
